# Near-infrared time-resolved spectroscopy reveals task-induced emotions by measuring cerebral blood oxygenation changes in the frontal pole during creative activity using a real object

**DOI:** 10.1016/j.ibneur.2025.06.009

**Published:** 2025-06-17

**Authors:** Yumi Oboshi, Kazuki Tamura, Yasuko Fukushi, Seiji Yamamoto

**Affiliations:** a Biomedical Instrumentation Laboratory, Research and Development in Photonics Technology, Institute of Photonics Medicine, Hamamatsu University School of Medicine, 1-20-1 Handayama, Chuo-ku, Hamamatsu 431-3192, Japan; b Medical School, Department of Health Sciences, Major in Rehabilitation Science, Occupational Therapy Program, Nagoya City University, 1 Kawasumi, Mizuho-cho, Mizuho-ku, Nagoya 467-8601, Japan; c Biomedical Photonics Research Division, Research Institute of Electronics, Shizuoka University, 3-5-1 Johoku, Chuo-ku, Hamamatsu 432-8011, Japan

**Keywords:** Creative activity, Positive emotion, Cerebral blood oxygenation, Frontal pole, Near-infrared time-resolved spectroscopy, Real object

## Abstract

Creative activities trigger enjoyable feelings, induce motivation, and are applied in clinical settings such as rehabilitation. Emotion and creativity are interrelated because they depend on a common neural network, with the prefrontal cortex playing a crucial role in both. Emotions affect creative thinking, and creative activities elicit emotions. Near-infrared spectroscopy (NIRS) provides a real-time assessment of emotion generated in a natural setting. Furthermore, near-infrared time-resolved spectroscopy (NIR-TRS) can measure brain activity that is less susceptible to extracerebral tissue. We measured oxyhemoglobin (Oxy-Hb) concentrations in the frontal pole, which is involved in emotion processing using NIR-TRS during creative and simple tasks utilizing real objects. Oxy-Hb concentrations in the frontal pole significantly increased during and after the creative task compared with the simple task. The autonomic function indices (heart rate and stress indices) were inversely correlated with the Oxy-Hb increase associated with the creative task performance, indicating that sympathetic nervous system hyperactivity did not cause this Oxy-Hb increase. A subjective survey revealed that positive emotions during the creative activity were significantly higher and correlated well with the increased Oxy-Hb level, indicating an increased frontal pole activity because of the enjoyability of the creative task. Our results indicate that NIR-TRS imaging can be employed for noninvasively measuring cerebral blood oxygenation changes in participants who experience various emotions during creative activities.

## Highlights

• NIR-TRS reveals greater Oxy-Hb increase during and after creative task.
• Autonomic function indices are inversely correlated to Oxy-Hb change.
• Creative task evokes greater positive emotions than simple task.
• After creative task Oxy-Hb increase is attributed to relaxed and positive emotions.
• NIR-TRS can assess emotional states during creative activities.

## Introduction

Creative activities popularly improve health and well-being and are applied as one of the therapeutic methods in the medical field such as occupational therapy and rehabilitation. Many people experience mental and emotional changes through creative activities. The neural underpinnings of creativity have rapidly become clear, and in recent years, the association between creative activity and emotion has also become increasingly clear.

The interaction between the default mode network (DMN) and the executive control network (ECN) is crucial as a neural network for creative thinking (Beaty et al., 2015). In particular, both emotional regulation and creative thinking mainly involve the medial prefrontal cortex and posterior cingulate cortex (Beaty et al., 2015, Beaty et al., 2016, Beaty et al., 2019, Khalil et al., 2019). In particular, creative thinking and emotion regulation share a common neural basis, and the prefrontal cortex (PFC) is involved in both processes (Khalil et al., 2025, Khalil et al., 2019). In recent empirical research, Khalil et al. investigated the effects of emotional induction (happy, neutral, sad) on ideational originality and the associated neural dynamics. They revealed that inducing a happy emotion may reduce neural activity and processing of rich information in working memory for exploring more original ideas through cognitive flexibility. Further, inducing a sad emotion may improve neural activity and increase coupling within the attention system to exploit and select fewer original ideas through cognitive persistence (Khalil et al., 2025). Response inhibition may mediate the association between emotional states and creative divergent thinking, and other mechanisms of this relationship are becoming more apparent (Khalil et al., 2023). Positive emotions improve cognitive flexibility and creative thinking. Emotions affect creativity, whereas creative activity evokes emotions and reinforces motivation, triggering feelings of accomplishment and pleasure through the reward system (dopamine pathway) (Khalil et al., 2019). The PFC, hippocampus, basal ganglia, cerebellum, and dopamine may promote novelty seeking in the creative process (Khalil and Moustafa, 2022).

Recent studies further demonstrated the essential role of the PFC in emotion and creativity using functional magnetic resonance imaging (fMRI), electroencephalography (EEG), and transcranial direct current stimulation (tDCS). For instance, fMRI studies have explored the important connection between amygdala-to-inferior frontal gyrus connectivity in creativity and humor processing (Chan et al., 2023). A network approach to localizing creativity in the brain across different creative domains, including music, writing, drawing, and divergent thinking, revealed that the brain circuit for creativity was defined by negative functional connectivity between coordinates activated by creativity tasks and the right frontal pole, indicating this region as the circuit hub. The authors speculate that frontal pole deactivation may reduce self-monitoring and release of disinhibited creative output or spontaneous improvisation (Kutsche et al., 2025). DMN may play a crucial role in internally directed cognition such as divergent thinking in the creative process and mind-wandering. Further, an EEG study demonstrated that the network was differentially recruited between divergent processes and mind-wandering (Bartoli et al., 2024). Several studies that used tDCS have presented evidence of the involvement of PFC in creativity, especially on the creative cognition side (Khalil et al., 2020) as well as the association between stress and creativity (Wang et al., 2022, Wang et al., 2023).

We commonly experience positive emotions such as enjoyment, pleasure, and a sense of accomplishment during and after the process of making things. Moreover, making things and engaging in creative activities have a good effect on mental health (Bone et al., 2023) and reduce stress (Martin et al., 2018). For example, Shiraiwa et al. (2020) reported that a relaxed-concentration state is achieved by focusing on a craft activity. In clinical rehabilitation settings, such as occupational therapy, these effects on the physical and mental benefits of creative activities are used as a helpful tool (Akiyama et al., 2021, Boersma et al., 2021, Liu et al., 2024, Pérez-Sáez et al., 2020).

When extracting emotions, natural stimuli such as movies, videos, and narrative stories are considered more appropriate for emotion research than are artificial stimuli generated for the experiments and presented on a monitor, because they elicit stronger emotions. The study of emotion in natural situations is a growing field of interest (Jääskeläinen et al., 2021, Saarimäki, 2021). Furthermore, real objects elicit different responses compared with images. In this case, real objects are tangible solids with which individuals can interact (Snow and Culham, 2021). Moreover, real objects have the advantage of enhancing recognition compared with images, are more memorable than images, and attract attention more easily than images (Snow and Culham, 2021). Recently, the evidence that both behavior and brain function differ between image proxies and that artificial two-dimensional (2-D) images of stimuli that are not actually present and real/tangible objects has been increasing (Snow and Culham, 2021). For example, the viewing of actual foliage plants elicited a greater prefrontal activation compared with a projected image of foliage plants (Igarashi et al., 2015). In addition, real snack foods were reported to increase willingness-to-pay compared with 2-D images of the same items (Romero and Snow, 2019). Regarding the brain response that is elicited during the execution of a craft activity, i.e., the actual making of things, Shiraiwa et al. (2020) examined changes in the frontal midline theta rhythm (Fmθ) and autonomic nervous responses, and revealed that a certain relaxed-concentration state is achieved by focusing on craft activities. Moreover, Orui et al. (2024) showed that parasympathetic activity, which represents a state of relaxation and dyadic physiological synchrony, increased when the same craft activity was performed in parallel groups. These findings suggest that examining brain function during a creative activity using real objects will allow the assessment of stronger emotions. In our study, a creative activity using a real object was selected as the task for the purpose of evoking positive emotions under natural conditions.

Near-infrared spectroscopy (NIRS) was selected for the assessment of the brain response to the emotions induced by the creative activity because it is capable of performing measurements in a more naturalistic environmental setting (Ferrari and Quaresima, 2012). NIRS is considered appropriate for exploring neuroscience in the everyday world (von Lühmann et al., 2021). Studies of brain responses in the real world, i.e., under natural settings and in daily life, have become even more important (Stangl et al., 2023). NIRS can employ a wider range of tasks (Pinti et al., 2018, Pinti et al., 2020). Furthermore, NIRS-based brain imaging is more suitable for conducting in realistic situations rather than in laboratory environments. For example, Burgess et al. (2022) measured the changes in brain activity in participants involved in prospective memory tasks while walking through a city. Furthermore, recently, some studies using NIRS for assessing brain activity during the real-world aesthetic evaluation of body movements have been conducted to investigate the extent to which embodied experience affects enjoyment of synchronized movement and the cortical mechanisms underlying these aesthetic judgments (Casale et al., 2024, Moffat and Cross, 2024).

Various major NIRS measurement techniques that include continuous-wave near-infrared spectroscopy (CW-NIRS), which uses continuously emitted light (Villringer and Chance, 1997), and near-infrared time-resolved spectroscopy (NIR-TRS), which uses short-pulsed lasers (Patterson et al., 1989), are commonly used for brain imaging. In near-infrared systems, picosecond pulses of light are emitted into the tissue, and the arrival times of the photons are measured at nearby detectors (Ban et al., 2022). NIR-TRS estimates the absorption coefficients and calculates the absolute hemoglobin concentration.

CW-NIRS studies have reported that emotional stimuli increase Oxy-Hb concentration (Boas et al., 2004, Kim et al., 2021). To date, many studies of emotion generation and emotion regulation have been conducted using a CW-NIRS (Bendall et al., 2016, Westgarth et al., 2021). However, the responses to emotional stimuli are inconsistent; while some studies found greater Oxy-Hb concentration for both positive and negative emotions than neutral ones, others showed decreased, no change, or changes dependent on emotional valence (Bendall et al., 2016, Westgarth et al., 2021). One explanation for this inconsistency may be the weakness of the emotional stimulus, because most of the stimuli used in those studies were 2D images. We thought that the 3D real object and the active creation elicited stronger emotions. Moreover, the assessment of emotionally evoked cerebral blood oxygenation changes using a CW-NIRS is likely affected by the oxygenation of the extracerebral tissues, such as the scalp. Therefore, we conjectured that NIR-TRS would provide a measurement less influenced by extracerebral tissues (Gunadi et al., 2014, Sato et al., 2007). Many CW-NIRS studies show that prefrontal cortex plays a crucial role in emotion generation and emotion regulation. However, to the best of our knowledge, studies using NIR-TRS brain imaging to assess emotions are lacking.

In the present study, we focused on frontal pole activation because positive emotions, such as happiness, activate the medial prefrontal cortex, including the frontal pole according to fMRI studies (Matsunaga et al., 2016, Sharot et al., 2007). Furthermore, the subjective humor sensation under the natural condition of watching a comedy movie has been reported to be associated with frontal pole activity, together with hippocampal activity (Iidaka, 2017). Therefore, we hypothesized that frontal pole activity would increase while performing an enjoyable creative task using a real object and that NIR-TRS can detect emotion-induced cerebral blood oxygenation in the frontal pole. To verify these hypotheses, using NIR-TRS of frontal pole, we compared Hb concentration changes in participants as they performed creative and simple tasks, and their responses to subjective emotion questionnaire.

## Experimental procedures

### Participants

Twenty-four healthy volunteers (11 men and 13 women) were enrolled in this study. All participants were right-handed and assessed using the Edinburgh Handedness Inventory (Oldfield, 1971). The subjects had normal or corrected-to-normal vision and no past or current neurological or psychiatric disorder history. The Ethics Committee of Hamamatsu University School of Medicine (Japan) approved this study, and all procedures complied with the Declaration of Helsinki. All participants provided written informed consent before enrollment. One male subject was excluded because of a motion artifact. Therefore, we analyzed twenty-three participants (10 men and 13 women; age range: 21–26 years; mean age: 22.2 ± 1.51years).

### Experimental protocol

**Fig. 1:**
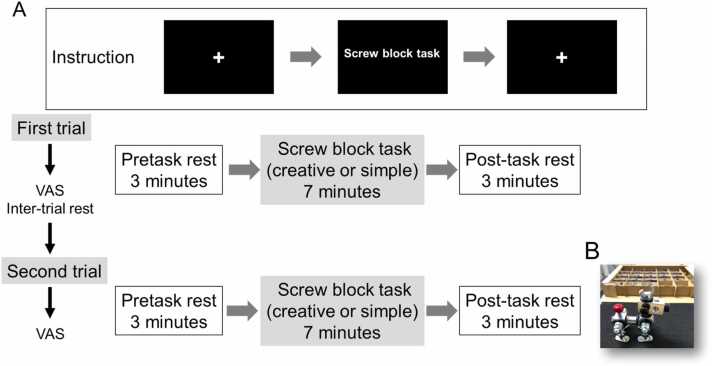
**Experimental protocol and content of activities performed by the participants during NIR-TRS measurement.** A. Instructions to the participants were provided through a display monitor; B. Example of a participant’s work. In the creative task, participants were instructed to make a “dog figure” by combining parts of the screw block referencing the construction manual.

Participants fitted with optode sensors on forehead (see NIR-TRS measurement section below) performed two types of tasks: creative and simple. For creative task, participants were instructed to spend 7 min making a dog figure “Taro” (Screw Block® assembly kit, Hashimoto Rashi Co., Hamamatsu, Japan) using an instruction manual (Akiyama et al., 2021). For simple, repetitive task, they were requested to spend 7 min, repeatedly, tightening and untightening. The participants had undergone before (pretask) and after (post-task) rest periods wherein they were asked to fix their gaze on a cross on the monitor screen for 3 min without thinking anything (Fig. 1). A display monitor located in front of the participants provided them instructions (Fig. 1). Throughout the experiment, participants were requested to move their heads and bodies minimally. The participants rested for 3 min before switching between the creative and simple tasks. Immediately following completion of a given task, participants answered questions regarding their feelings and state of mind (emotions) during the task; a visual analogue scale (VAS), described below, was used for this analysis (Sung and Wu, 2018).

### Subjective investigation of emotions using VAS

To analyze the emotions of the participants after the completion of each task, we created an original questionnaire using VAS (minimum value: 0, maximum value: 100) and requested participants to respond to the following 7 items: Q1, Enjoyment (the higher the number, the more “I enjoyed” [when doing that task]); Q2, Concentration (the higher the number, the more “I could concentrate” [when doing that task]); Q3, Difficulty (the higher the number, the more “I felt difficult” [in doing that task]); Q4, Motivation (the higher the number, the more “I was motivated” [when doing that task]); Q5, Arousal (the larger the number, the “more awake I felt” [in the task]; the smaller the number, the “more sleepy I felt” [in the task]); Q6, Lack of Fatigue (the larger the number, the “less tired I felt” [when doing the task]; the smaller the number, the “more tired” [when doing the task]); and Q7, Like/Dislike (the larger the number, the “more I like” [the task]). The questionnaire using the VAS was administered immediately after each post-task rest, to avoid the bias caused by memory errors.

### Hemodynamics measurement using NIR-TRS

**Fig. 2:**
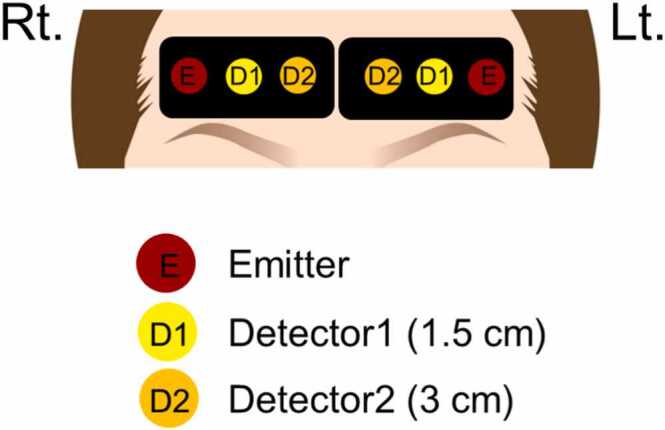
**Position of the optodes used for NIR-TRS imaging of frontal pole activity.** The distance between emitter and detector 1 is 1.5 cm (Detector 1 detects hemoglobin concentration changes at a depth of 1.5 cm from extracerebral tissues); the distance between emitter and Detector 2 is 3 cm (Detector 2 detects hemoglobin concentration changes in the brain parenchyma at a depth of 3 cm from the scalp).

We placed the optode sensors on the forehead of participants–on the right and left sides of the midline based on FpZ as a reference point, according to the international 10/20 system for EEG (Fig. 2). We used TRS-41 (Hamamatsu Photonics K.K., Hamamatsu, Japan) with three wavelengths (Ch1: 763, 802, and 835 nm, Ch2: 762, 800, and 837 nm) to examine the Hb concentration change of the participants. This system used the time-correlate single photon counting method to measure the temporal function of the sample. The intensity of light in a time domain along with the time domain photon diffusion equation enabled data analysis (Ohmae et al., 2006, Patterson et al., 1989). The sampling rate of the recording was 0.33 Hz.

### Heart rate and P–P interval measurement

To investigate the response of the autonomic/sympathetic nervous system, electrocardiograms (ECG) were recorded simultaneously with NIR-TRS measurement. The heart rate and P–P interval (the time between successive P waves in the ECG) of the participants during both types of tasks were recorded using the 16-channel recording system MP160 (BIOPAC systems, Inc., Goleta, CA, USA) (Arquilla et al., 2020).

## Statistical analysis of data

### Behavioral data analysis

For all participants, VAS scores for each question were averaged and compared between the two conditions. The Shapiro–Wilk test was used to examine the normality distribution of VAS scores in each task. A paired *t*-test was used to compare Enjoyment and Like/Dislike, because their VAS scores were normally distributed, for both task conditions. A Wilcoxon matched-pairs signed rank test was used for Arousal, Concentration, Difficulty, Motivation, and Lack of Fatigue, because their VAS scores were not normally distributed, for one or none of the tasks.

### Analysis of task-induced hemodynamic changes

For NIR-TRS analysis, the measured photon counting data were fitted to the photon diffusion equation over a time window. The solution of a semi-infinite mode for the reflectance mode (Patterson et al., 1989) convolved the instrumental response function was fitted into the observed temporal profiles obtained from the NIR-TRS systems. This determined the absorption coefficient and the reduced scattering coefficient at each wavelength. Then, Oxy-Hb, deoxyhemoglobin (Deoxy-Hb), and total hemoglobin (Total-Hb) were calculated using the least squares method (Ohmae et al., 2006).

To analyze the task-induced hemodynamic change, the values of Oxy-Hb, Deoxy-Hb, and Total-Hb of all subjects (n = 23) during each condition were averaged. Then, we calculated the rate of change (%) of each Hb concentration value because the baseline concentrations varied among the participants. Each data point value was divided by the average of ten data points corresponding to approximately 33 s just before the pretask rest started. Because the duration of the creative activity was relatively long (7 min), the transition of the brain activation associated with emotion was predicted during the task. Therefore, the averaged change rate was calculated for 13 segmental periods, 1 min in duration (pretask rest1, pretask rest2, pretask rest3, task1, task2, task3, task4, task5, task6, task7, post-task rest1, post-task rest2, post-task rest3) for each task (creative, and simple). We analyzed two channels to detect brain activity: ch1 (right hemisphere) and ch2 (left hemisphere).

Cohen’s d was calculated as an indicator of the effect size, and based on the effect size calculated; the statistical power was calculated using G*Power3.1 (Faul et al., 2009). The Shapiro–Wilk test confirmed that the hemoglobin change values for almost the entire period were normally distributed.

Statistically, we analyzed the rate of change of Oxy- and Deoxy-Hb in each period using two-way repeated measures of analysis of variance [task condition (2) * period (13)]. Paired *t*-tests were used to compare the two conditions (simple vs. creative). A significance level was set at p < 0.05 using SPSS software for Windows (version 25, IBM Corp., Armonk, NY, USA).

### Correlation analysis between Oxy-Hb and autonomic function indices

We analyzed the mean heart rate as an index of autonomic nervous function using AcqKnowledge (version 5, BIOPAC Systems, Inc., Goleta, CA, USA) Balconi and Maria Elide Vanutelli (2017) after visual inspection was performed and artifacts that appeared to be clearly off the baseline were removed. In the correlation analysis between cerebral blood oxygenation data and ECG data, two additional participants were excluded from the 23 participants, because their ECG data contained a lot of noise caused by body movement. The mean heart rate of 21 participants was calculated for every 13 periods, and the correlation between the Oxy-Hb concentration change rate and heart rate was calculated.

The low-frequency/high-frequency ratio (LF/HF value), a stress index obtained from the AcqKnowledge software (Blanch et al., 2013), was calculated for each of the 13 periods, and the correlation between the LF/HF value and Oxy-Hb concentration was calculated. In the correlation analysis between heart rate, LF/HF values and Oxy-Hb values, outliers were examined and excluded for each period for each channel using an outlier detection method built in GraphPad Prism7 software (GraphPad Software Inc., La Jolla, CA, USA). The Shapiro–Wilk test was then performed; subsequently, normally distributed values were analyzed using Pearson correlation coefficient and non-normally distributed values were analyzed using Spearman correlation coefficient.

### Correlation analysis between Oxy-Hb and subjective investigation VAS scores

To clarify the actual cause of Oxy-Hb increase observed after the completion of the given task, the Oxy-Hb increase rate in the immediate post-task rest period (1 min duration) was calculated. Correlations between Oxy-Hb change rate and subjective emotions VAS scores were analyzed using Pearson’s correlation coefficient for creative and simple task conditions (n = 23, both conditions per subject).

## Results

### Behavioral data confirmed emotional responses during creative task performance

**Fig. 3:**
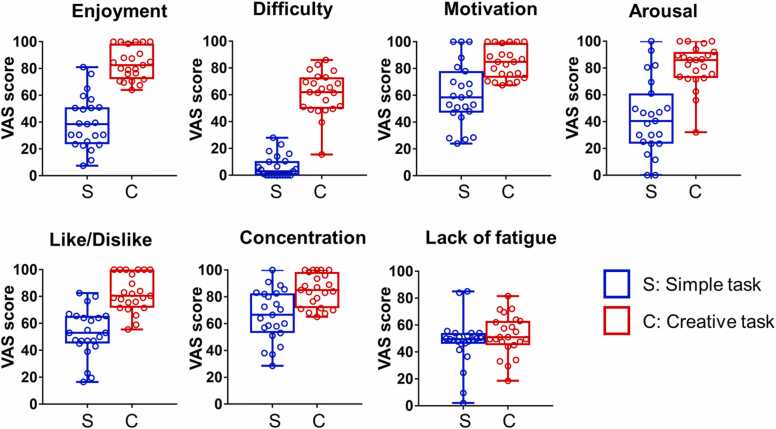
**Participants’ psychological state after performing each screw block task.** The box and whisker plot shows the distribution of VAS scores of 23 participants. The boxes range from the 25–75 %. Red and blue open circles represent individual data points for the creative and simple tasks, respectively.

Enjoyment and Like/Dislike were normally distributed in both groups, so a paired *t*-test was performed. Arousal, concentration, motivation, difficulty, and lack of fatigue were compared between groups using Wilcoxon matched-pairs signed rank test because one of the groups was not normally distributed. VAS scores for positive emotions were significantly greater subsequent to creative task compared to that subsequent to simple task (Table 1, Fig. 3). VAS score for “Lack of fatigue” was not significantly different between the two tasks (Table 1, Fig. 3). The differences in VAS scores indicate that the participants felt the creative tasks were enjoyable, likeable and challenging. Further, they were awake, concentrated and motivated. The participants did not score differently for “feeling less tired” in the two tasks, suggesting that the participants felt the same degree of fatigue following the creative and simple tasks.

**Table 1:** Differences between conditions in VAS scores in 23 participants.

	Simple	Creative	t or W	df	p
	Mean ± SD	Mean ± SD			
Enjoyment	39.61 ± 19.61	83.57 ± 11.95	t: 9.701	22	< 0.0001^***^
Arousal	43.17 ± 27.7	81.17 ± 16.04	W: 231	-	< 0.0001^***^
Like/Dislike	52.72 ± 17.64	82.48 ± 13.76	t: 6.498	22	< 0.0001^***^
Concentration	66.26 ± 19.11	85.04 ± 12.14	W: 202	-	0.0004^***^
Difficulty	6.283 ± 8.301	60.96 ± 15.91	W: 276	-	< 0.0001^***^
Motivation	60.28 ± 23.17	84.17 ± 11.75	W: 198	-	< 0.0001^***^
Lack of fatigue	47.37 ± 18.08	52.35 ± 14.91	W: 83	-	0.2139

The data is represented as mean ± standard deviation. t: t value of paired *t*-test. W: sum of signed ranks of Wilcoxon matched-pairs signed rank test. n = 23, ^***^p < 0.001.

### Oxy-Hb increases subsequent to creative task

**Fig. 4:**
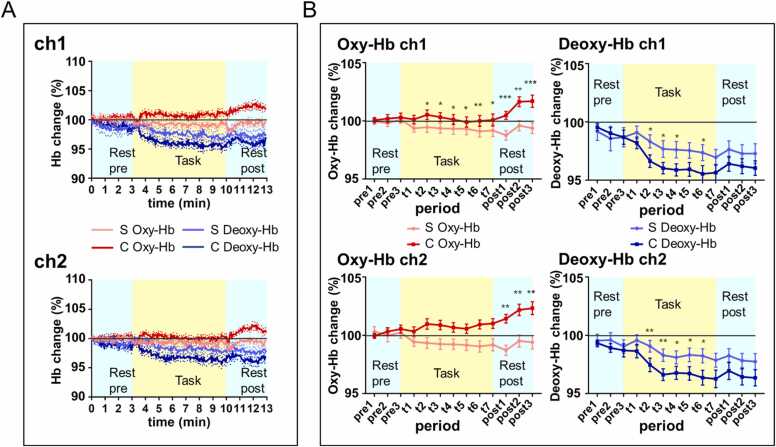
**Differences in Oxy-Hb and Deoxy- Hb concentration changes between the creative and simple tasks.** A. Average waveform of Oxy-Hb and Deoxy-Hb change of 23 participants. Red and blue lines indicate Oxy-Hb and Deoxy-Hb changes in the creative task, and pink and light blue lines indicate Oxy-Hb and Deoxy-Hb changes in the simple task. “S” indicates the simple task and “C” indicates the creative task. The standard error is indicated by dashed lines; B. Mean Oxy-Hb and Deoxy-Hb changes over time in 13 periods (n = 23). Red and blue lines indicate Oxy-Hb and Deoxy-Hb changes in the creative task, pink and light blue lines indicate Oxy-Hb and Deoxy-Hb change in the simple task. “S” indicates the simple task and “C” indicates the creative task. Pre1, pre2 and pre3 represents pretask rest1, pretask rest2 and pretask rest3. Post1, post2 and post3 represents post-task rest1, post-task rest2 and post-task rest3. Significant differences in Hb change between the two conditions over time were revealed by two-way repeated measures analysis of variance and paired *t*-test (The error bars represent the standard error; *p < 0.05, ^**^p < 0.01, ^***^p < 0.001).

The ch1 and ch2 waveforms for changes in averaged Oxy- and Deoxy- Hb concentrations (n = 23) exhibited the following trend: both ch1 and ch2 waveforms demonstrated that creative and simple tasks evoked different Oxy-Hb responses from their corresponding pretask rest period levels. While simple task did not induce Oxy-Hb level increase (from pretask rest period levels), creative task induced a significant increase in Oxy-Hb level compared to corresponding pretask rest period levels. Further, Deoxy-Hb decreased during the simple and creative tasks compared to their corresponding pretask period levels (Fig. 4). We examined statistically whether there were differences in conditions (over time) for the mean hemoglobin changes of the observed in the 23 participants. The Mauchly’s sphericity test for Oxy-Hb and Deoxy-Hb for all 13 periods in ch1 and ch2 revealed that sphericity could not be assumed for our data. Therefore, Greenhouse–Geisser’s correction was applied for a main effect [period (pretask rest1, pretask rest2, pretask rest3, task1, task2, task3, task4, task5, task6, task7, post-task rest1, post-task rest2, and post-task rest3)] and interaction [condition (simple, creative) * period (pretask rest1, pretask rest2, pretask rest3, task1, task2, task3, task4, task5, task6, task7, post-task rest1, post-task rest2, and post-task rest3)]. For Oxy-Hb change in ch1, a main effect [condition] was significant, a main effect [period] was not significant, and the interaction between [condition] and [period] was significant (Table 2); in ch2, a main effect [condition] was significant, a main effect [period] was not significant, and the interaction between [condition] and [period] was significant (Table 2). For Deoxy-Hb change, in ch1, the main effect [condition] was not significant, the main effect [period] was significant and the interaction between [condition] and [period] was significant (Table 2). In ch2, the main effects [condition] and [period] were significant, and the interaction between [condition] and [period] was not significant (Table 2).

**Table 2:** Differences between conditions in hemoglobin levels over time in 23 participants.

	Oxy-Hb				Deoxy-Hb			
	ch1		ch2		ch1		ch2	
	F	p	F	p	F	p	F	p
main effect [condition]	7.64	0.011*	4.66	0.042*	2.23	0.15	4.70	0.041*
main effect [period]	1.51	0.232	2.23	0.113	10.32	< 0.001^***^	7.99	< 0.001^***^
interaction [condition* period]	10.51	< 0.0001^***^	5.32	< 0.001^***^	3.17	0.022*	1.55	0.204

Main effect of condition (2: simple vs. creative) and period (13: pretask rest1/pretask rest2/pretask rest3/t1/t2/t3/t4/t5/t6/t7/post-task rest1/post-task rest2/post-task rest3), and their interaction effect by two-way repeated measures of analysis of variance, along with statistical information (F-statistic, p-value). *p < 0.05, ^***^p < 0.001.

A paired *t*-test was used to examine the difference in the mean rate of change of Oxy-Hb or Deoxy-Hb between the simple and creative task performances in each period. Creative task, induced significantly greater rate of increase in Oxy-Hb in ch1 in t2 to post3 periods. Meanwhile, it induced a significantly greater rate of increase in Oxy-Hb in ch2, in the post1/ post2/ post3 periods (Table 3). Compared to the simple task, the creative task induced a significantly greater rate of decrease of Deoxy-Hb in ch1 in the t2/ t3/ t4/ t6 periods and a significantly greater rate of decrease of Deoxy-Hb in ch2 in t2 to t6 periods (Table 4). In conclusion, compared to the simple task, the creative task induced a significantly greater rate of Oxy-Hb increase in—(a) the right frontal pole during the task and in the post-task rest periods; and (b) the left frontal pole during the post-task rest periods. Further, compared to the simple task, the creative task induced a significantly greater rate of decrease in Deoxy-Hb in the right and left frontal poles during the task. Further, creative task induced the Oxy-Hb supply to exceed the Deoxy-Hb increase in frontal pole.

**Table 3:** Comparisons between the two conditions (simple vs. creative) of the Oxy-Hb % change rate.

	ch1						ch2					
period	C: Creative S: Simple Mean ± SD	t	df	p	effect size (d)	power	C: Creative S: Simple Mean ± SD	t	df	p	effect size (d)	power
pretask rest1	C: 1.000 ± 0.010 S: 1.003 ± 0.020	0.83	22	0.417	0.24	0.20	C: 1.001 ± 0.011 S: 1.000 ± 0.013	−0.25	22	0.808	0.08	0.07
pretask rest2	C: 1.003 ± 0.016 S: 1.000 ± 0.027	−0.58	22	0.565	0.15	0.11	C: 1.002 ± 0.017 S: 0.998 ± 0.014	−0.97	22	0.343	0.25	0.21
pretask rest3	C: 1.005 ± 0.016 S: 1.002 ± 0.022	−0.70	22	0.493	0.17	0.12	C: 1.003 ± 0.018 S: 1.001 ± 0.015	−0.49	22	0.629	0.11	0.08
t1	C: 1.003 ± 0.019 S: 0.994 ± 0.018	−1.67	22	0.109	0.49	0.61	C: 1.001 ± 0.017 S: 0.994 ± 0.016	−0.16	22	0.118	0.46	0.56
t2	C: 1.010 ± 0.022 S: 0.993 ± 0.023	−2.77	22	0.011*	0.74	0.92	C: 1.005 ± 0.020 S: 0.995 ± 0.022	−2.03	22	0.055	0.51	0.65
t3	C: 1.009 ± 0.019 S: 0.992 ± 0.022	−2.60	22	0.016*	0.79	0.95	C: 1.003 ± 0.019 S: 0.994 ± 0.020	−1.87	22	0.075	0.49	0.61
t4	C: 1.007 ± 0.020 S: 0.992 ± 0.022	−2.32	22	0.03*	0.70	0.89	C: 1.001 ± 0.021 S: 0.993 ± 0.022	−1.44	22	0.165	0.37	0.40
t5	C: 1.006 ± 0.019 S: 0.992 ± 0.022	−2.29	22	0.032*	0.68	0.88	C: 0.999 ± 0.021 S: 0.993 ± 0.025	−1.06	22	0.30	0.25	0.21
t6	C: 1.009 ± 0.018 S: 0.991 ± 0.023	−3.23	22	0.004**	0.89	0.98	C: 1.000 ± 0.022 S: 0.991 ± 0.023	−1.71	22	0.102	0.39	0.43
t7	C: 1.010 ± 0.020 S: 0.992 ± 0.025	−2.41	22	0.025*	0.81	0.96	C: 1.001 ± 0.025 S: 0.992 ± 0.025	−1.55	22	0.135	0.37	0.40
post-task rest1	C: 1.014 ± 0.018 S: 0.987 ± 0.021	−4.23	22	< 0.001***	1.41	1.00	C: 1.005 ± 0.016 S: 0.988 ± 0.020	−3.03	22	0.006**	0.94	0.99
post-task rest2	C: 1.022 ± 0.024 S: 0.995 ± 0.025	−3.62	22	0.002**	1.09	1.00	C: 1.016 ± 0.020 S: 0.996 ± 0.021	−3.01	22	0.006**	0.98	0.99
post-task rest3	C: 1.023 ± 0.026 S: 0.994 ± 0.028	−4.16	22	< 0.001***	1.08	1.00	C: 1.017 ± 0.025 S: 0.994 ± 0.022	−3.27	22	0.003**	0.98	0.99

The data is represented as mean ± standard deviation, along with t-statistic, effect size (Cohen’s d), and the achieved power computed as a post hoc analysis. n = 23, * p < 0.05, ** p < 0.01, *** p < 0.001.

**Table 4:** Comparisons between the two conditions (simple vs. creative) of Deoxy-Hb % change rate.

	ch1						ch2					
period	C: Creative S: Simple Mean ± SD	t	df	p	effect size (d)	power	C: Creative S: Simple Mean ± SD	t	df	p	effect size (d)	power
pretask rest1	C: 0.995 ± 0.014 S: 0.992 ± 0.037	−0.58	22	0.571	0.13	0.09	C: 0.993 ± 0.011 S: 0.995 ± 0.026	0.41	22	0.687	0.10	0.07
pretask rest2	C: 0.990 ± 0.023 S: 0.986 ± 0.050	−0.53	22	0.604	0.12	0.09	C: 0.989 ± 0.018 S: 0.996 ± 0.030	1.22	22	0.237	0.27	0.24
pretask rest3	C: 0.987 ± 0.029 S: 0.987 ± 0.040	−0.01	22	0.995	0.00	0.05	C: 0.987 ± 0.027 S: 0.990 ± 0.028	0.59	22	0.564	0.12	0.09
t1	C: 0.982 ± 0.024 S: 0.991 ± 0.026	1.36	22	0.188	0.36	0.38	C: 0.986 ± 0.028 S: 0.996 ± 0.022	1.66	22	0.111	0.37	0.40
t2	C: 0.966 ± 0.026 S: 0.983 ± 0.027	2.33	22	0.029*	0.64	0.83	C: 0.975 ± 0.028 S: 0.991 ± 0.024	2.93	22	0.008**	0.62	0.81
t3	C: 0.960 ± 0.024 S: 0.977 ± 0.034	2.12	22	0.046*	0.56	0.73	C: 0.966 ± 0.024 S: 0.983 ± 0.026	3.31	22	0.003**	0.67	0.87
t4	C: 0.959 ± 0.025 S: 0.976 ± 0.034	2.28	22	0.032*	0.58	0.76	C: 0.968 ± 0.027 S: 0.981 ± 0.027	2.46	22	0.022*	0.49	0.61
t5	C: 0.959 ± 0.028 S: 0.975 ± 0.032	1.91	22	0.069	0.53	0.68	C: 0.967 ± 0.028 S: 0.983 ± 0.028	2.47	22	0.022*	0.57	0.74
t6	C: 0.955 ± 0.034 S: 0.974 ± 0.033	2.21	22	0.038*	0.55	0.71	C: 0.964 ± 0.030 S: 0.982 ± 0.029	2.63	22	0.015*	0.63	0.82
t7	C: 0.956 ± 0.033 S: 0.970 ± 0.033	1.55	22	0.135	0.40	0.45	C: 0.963 ± 0.036 S: 0.979 ± 0.028	1.91	22	0.07	0.49	0.61
post-task rest1	C: 0.964 ± 0.030 S: 0.976 ± 0.035	1.30	22	0.207	0.38	0.41	C: 0.970 ± 0.036 S: 0.983 ± 0.029	1.49	22	0.15	0.40	0.45
post-task rest2	C: 0.962 ± 0.031 S: 0.973 ± 0.037	1.08	22	0.292	0.32	0.31	C: 0.964 ± 0.033 S: 0.978 ± 0.029	1.50	22	0.148	0.45	0.54
post-task rest3	C: 0.960 ± 0.030 S: 0.973 ± 0.040	1.32	22	0.202	0.36	0.38	C: 0.964 ± 0.033 S: 0.977 ± 0.033	1.42	22	0.17	0.42	0.49

The data is represented as mean ± standard deviation, along with t-statistic, effect size (Cohen’s d), and the achieved power computed as a post hoc analysis. n = 23, * p < 0.05, ** p < 0.01.

### Association between brain activation during creative tasks, autonomic responses, and subjective investigation

**Fig. 5:**
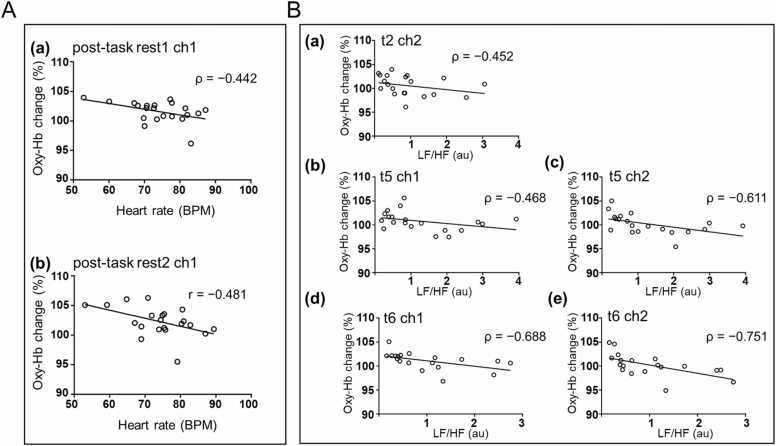
**Association between brain activation and autonomic nervous system indices.** A. Association between heart rate and brain activation after the completion of the task: Heart rate was significantly inversely correlated with Oxy-Hb increase during the first minute after the end of the task: (a) ch1: n = 21, p = 0.0451, Spearman correlation coefficient; (b) ch2: n = 21, p = 0.0272, Pearson correlation coefficient; BPM, beats per minute; B. Association between stress indices (LF/HF) and brain activation, mainly during the latter half of the task:(a) t2: ch2, n = 21, p = 0.04, Spearman correlation coefficient; (b) t5: ch1, n = 20, p = 0.038, Spearman correlation coefficient; (c) t5: ch2, n = 20, p = 0.004, Spearman correlation coefficient; (d) t6: ch1, n = 19, p = 0.001, Spearman correlation coefficient; and (e) t6: ch2, n = 19, p = 0.0002, Spearman correlation coefficient.

Creative task induced an Oxy-Hb increase in ch1 and ch2, which began in latter half of the task and continued in to the post-task rest periods. To clarify the reason for this, we analyzed the association of the brain activation with autonomic responses (heart rate, LF/HF values) and subjective VAS scores. Oxy-Hb increase rate in ch1 was inversely correlated with heart rate during the creative task, during post1 (post-task rest 1 min following task completion) and post2 (post-task rest period 2–3 min following task completion) (post1: n = 21, ρ = −0.442, p = 0.045, Spearman, post2: n = 21, r = −0.481, p = 0.027, Pearson) (Fig. 5). This meant that a lower heart rate was associated with a greater right frontal pole Oxy-Hb increase during post-task rest periods.

Furthermore, correlations between LF/HF values and the Oxy-Hb increase rate were analyzed. Significant inverse correlations were observed for t5 (n = 20, ρ = −0.468, p = 0.038, Spearman) and t6 (n = 19, ρ = −0.688, p = 0.001, Spearman) in ch1 and t2 (n = 21, ρ = −0.452, p = 0.04, Spearman), t5 (n = 20, ρ = −0.611, p = 0.004, Spearman) and t6 (n = 19, ρ = −0.751, p = 0.0002, Spearman) in ch2 in the creative task (Fig. 5). Smaller stress values were associated with a greater increase in Oxy-Hb in the right and left frontal poles, indicating that the Oxy-Hb increase, mainly in the latter half of the task, was caused by factors other than stress. The finding that lower values of heart rate and LF/HF were associated with greater brain activation indicated that the increase in Oxy-Hb, mainly in the latter half of the task and post-task rest, was because of reasons other than sympathetic hyperactivity.

**Fig. 6:**
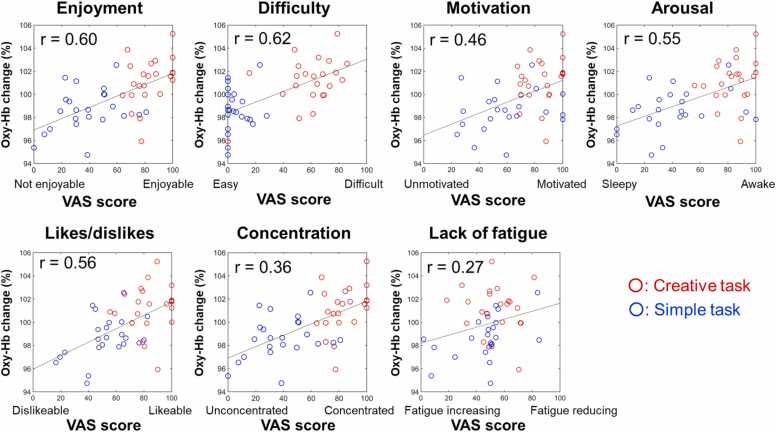
**Relationship between Oxy-Hb change and psychological state after completion of the creative task.** Mean 1-min Oxy-Hb change and VAS scores immediately after the end of the task. The VAS score is plotted on the horizontal axis and the Oxy-Hb change (the value of post1 is set to 100 %) on the vertical axis. The red open circle represents the creative task, and the blue open circle represents the simple task, r represents the correlation coefficient.

To further examine if the continued increase in Oxy-Hb in the post-task rest was an emotional response induced by the task execution or not, correlations were examined between the average 1-min Oxy-Hb change rate and VAS scores immediately after the end of the task. Peason’s correlation coefficient revealed that the change in Oxy-Hb concentration was strongly correlated with five metrics: Enjoyment (n = 23, r = 0.60, p = 0.0000), Difficulty (n = 23, r = 0.62, p = 0.0000), Motivation (n = 23, r = 0.46, p = 0.0011), Arousal (n = 23, r = 0.55, p = 0.0001), and Like/Dislike (n = 23, r = 0.56, p = 0.0000). Greater feeling of enjoyment, difficulty, arousal, high motivation, and liking correlated with larger Oxy-Hb change. Rate of Oxy-Hb concentration change was weakly correlated with the “concentration” VAS score (n = 23, r = 0.36, p = 0.0148). “Lack of fatigue” VAS score was not correlated with Oxy-Hb change rate (n = 23, r = 0.27, p = 0.0695) (Fig. 6). Thus, the Oxy-Hb increase during the rest period immediately following task completion may reflect positive feelings such as enjoyment, wakefulness, liking, etc., in addition to finding the task challenging.

## Discussion

### Creative activity induces frontal pole activation

Herein, we measured frontal pole activation using NIR-TRS —a noninvasive and more rigorous method compared to conventional CW-NIRS—to confirm if creative activity using a real object elicits greater cerebral oxygenation level compared with simple activity during and after the task. Furthermore, our subjective investigation of participant’s feelings demonstrated that they had positive emotions during the performance of the creative task. Our results indicate that brain requires a greater oxygen supply when engaged in creative activity. A previous study showed that subjects performing highly creative activities demonstrate greater brain activity in multiple brain regions, including the frontal pole (Chávez-Eakle et al., 2007), and our result is consistent with this finding.

Regarding brain network during the creative thinking, a functional network connectivity and the cooperation of two networks (i.e., DMN and ECN) are crucial (Beaty et al., 2015, Beaty et al., 2016, Beaty et al., 2019, Khalil et al., 2019). Within the PFC, the frontal pole is a part of the DMN and the dorsolateral PFC (DLPFC) is included in the ECN. The frontal pole coordinates two cognitive processes, consisting of stimulus-oriented cognition, which directs attention to external stimuli, and stimulus-independent cognition, which involves spontaneous and internal thought and memory operations (Burgess et al., 2007). Stimulus-independent cognition is the very core of creativity and supports creative thinking, in which information is retrieved from memory, with the most appropriate responses being selected to satisfy specific criteria such as originality and appropriateness (Boccia et al., 2015). Stimulus-independent cognition caused frontal pole activation during the current creative activity, and the frontal pole may be involved as part of the DMN. The creative task utilized in this study did not require flexible thinking or novelty. The completed form and creation process were predefined and clear; thus, a high creative thinking level was not required. Hence, it was closer to a craft activity that is frequently used in the medical setting, as it were, a creative problem-solving task. Moreover, self-relevance activated the frontal pole, which is a part of the DMN (Andrews-Hanna et al., 2010, Benoit et al., 2010, D'Argembeau et al., 2007). Therefore, the sensation of making things by oneself during the creative activity and the enjoyment it brings may activate the frontal pole. The self-reflective thinking that occurs during creative activity and the subjective experience of creation may have activated the DMN.

The effects of creative activities on neuroplasticity and the autonomic nervous system are known, and thus, they are frequently used therapeutically. Creative activities, such as musical, visuospatial, and kinesthetic activities, improve the development of new neural connections (Khalil and Demarin, 2024). Executive functions are improved through creative activities, and enhanced executive function promotes the brain plasticity effect (Khalil and Demarin, 2024). Promoting functional brain compensation and neuroplasticity through creative activities may be an effective nonpharmacological treatment for individuals with brain injury and neurodegenerative diseases such as dementia.

### Assessment of the emotion induced by a real object in a natural setting

Several studies have investigated neural activity during emotional processing using NIRS (Hoshi et al., 2011, Minematsu et al., 2018, Ozawa et al., 2014, Tupak et al., 2014). Most of them were passive tasks, such as watching images; therefore, the brain activation was considered to be relatively modest. In the current study, we hypothesized that the activity using a real object would elicit stronger emotions; in fact, frontal pole activity was observed. In particular, because the participants used their own hands to create the figure using a variety of real components, it is assumed that the self-relevance elicited a greater emotion, as well as changes in brain activity. In recent years, studies of emotion using natural stimuli have been attracting attention. Although NIRS does not allow whole-brain analysis, it is possible to examine changes in brain activation in a specific area if the area involved is clear in advance. When making things, the sitting position is the usual posture. Because it is easier to wear compared with EEG, NIRS measurements are suitable for the evaluation of emotional states during creative activities in daily life. In particular, NIR-TRS is more suitable for emotional research than is CW-NIRS because it is less susceptible to the effects of extracerebral tissues; however, in terms of portability, it is currently less portable than is CW-NIRS. Most of the NIR-TRS apparatuses reported to date are relatively large and are not suitable for portable use. Currently, portable NIR-TRS systems are available (Ban et al., 2022). As the importance of emotion measurements in natural environments and in everyday situations is expected to increase in the future, device portability is important for this process (Stangl et al., 2023). It is likely that more emotion research will be carried out using the portable NIR-TRS system. However, it may take some time before portable NIR-TRS is widely used because of cost issues.

Herein, we stimulated brain activity using a creative task involving use of metal screws placed on a desk. Thus, we could not use fMRI or positron emission tomography to study brain activity. We used real objects, i.e., three-dimensional objects, because they evoke greater brain activity in comparison to two-dimensional images (Marini et al., 2019). Furthermore, recall and recognition types of cognitive function are performed significantly better when real objects are used compared to when colored photographs or line drawings are used (Snow et al., 2014). In this study, we successfully used NIR-TRS to detect brain activity during the creative task performance, indicating that NIR-TRS may be appropriate for examining brain activity in practical situations.

Our subjective investigation, expectedly, revealed that the participants liked the creative task more than the given simple task. Further, they felt more enjoyment, were more awake and focused, and were more challenged when performing it. Moreover, although the given creative task was slightly less tiring than the simple task, neither task significantly affected the fatigue scores. We hear similar empirical impressions and feedbacks from participants performing creative tasks in rehabilitation/therapeutic recreation settings.

### Relationship between autonomic nervous system response and brain activation during creative tasks

Moreover, creative activities affect both the sympathetic (active and aroused) and parasympathetic (relaxed and restorative) nervous systems. Regarding physical activities, in addition to the autonomic nervous system, neuromodulation mechanisms, such as the hypothalamic–pituitary–adrenal axis and the brain-visceral coupling caused the temporary activation of the sympathetic nervous system, resulting in a flow state of increased attention and concentration (Khalil and Demarin, 2024). Music and art activities activate the parasympathetic nervous system and alleviate anxiety, depression, and stress reactions.

In this study, we measured the heart rate and variability in heart rate as indicators of autonomic nervous functions. The heart is dually controlled by the sympathetic and parasympathetic nerves, and heart rate increases with sympathetic activity and decreases with parasympathetic activity (Robinson et al., 1966). Emotional states change heart rate. A significant reduction of heart rate is observed in response to gratitude intervention compared to that observed in response to resentment intervention (Kyeong et al., 2017). Heart rate increases with sympathetic hyperactivity. Herein, when performing the creative task, the degree of Oxy-Hb increase was smaller when heart rate of the post-task rest phase was higher. Positive and negative emotions increase heart rate (Kreibig, 2010), and increased stress is associated with increased heart rate (Hakimi and Setarehdan, 2018). Studies of brain response to tactile sensations and natural sounds using NIR-TRS or CW-NIRS have reported decreased Oxy-Hb and heart rate in circumstances that are supposed to produce a relaxed state (Ikei et al., 2017, Jo et al., 2019). Conversely, herein we demonstrate that increase in brain activation correlates with a decrease in heart rate following the completion of the creative task. Thus, the increase in brain activation, observed in this study, is not because of sympathetic hyperactivity.

Heart rate variability (HRV) is a stress indicator (Immanuel et al., 2023). LF/HF ratio represent the balance between sympathetic and parasympathetic activities, with high and low values indicating a predominance of sympathetic activity and parasympathetic activity, respectively (Pagani et al., 1986, Pagani et al., 1984). When performing the creative task, the Oxy-Hb increase and LF/HF values in the latter half of the task were inversely correlated (period t5 and t6). Thus, low stress is associated with a greater degree of brain activation. Furthermore, LF/HF values are increased by stress (Kim et al., 2018, Sloan et al., 1994). Prefrontal oxygenation level increases with mental stress tasks (Murayama et al., 2016, Nagasawa et al., 2020, Tanida et al., 2007) and decreases with mental relaxation (Yasui et al., 2010). In a previous study that examined the relaxing effects of natural sounds and tactile sensations by examining LF/HF values and brain activation, it was reported that prefrontal activation and LF/HF values decreased in the supposedly relaxed state (Ikei et al., 2017, Ikei et al., 2018, Jo et al., 2019). In contrast, in this study, prefrontal activation continued from the latter half of the task to the end of the task; the greater the stress index in that period, the smaller the activation tended to be. Therefore, we surmised that the continued increase in prefrontal oxygenation level is because of factors other than sympathetic hyperactivity like stress state. This holds true only if LF/HF values represent a balance between sympathetic and parasympathetic activity. If LF values do not reflect sympathetic activity alone, one cannot assert that high LF/HF values indicate a state of high sympathetic activity and stress (Billman, 2013, Gullett et al., 2023). However, clarifying this, is beyond the scope of this study.

### Continued brain activation during post-task rest period

Herein, when performing the creative task, a further Oxy-Hb increase was observed from the latter half of the task extending in to the post-task rest period. Increased Oxy-Hb after the end of task has also been reported in previous CW-NIRS studies on cognitive function and emotional processing (Butti et al., 2009, León-Carrión et al., 2007). An increment in subjective arousal leads to DLPFC activation, which persists after stimulus cessation; and further, a lasting poststimulus DLPFC activation occurs during processing of arousal caused by visual affective stimuli (León-Carrión et al., 2007). Moreover, our studies have shown that poststimulus activation is associated with VAS scores for emotional states such as enjoyment, arousal, and liking/disliking. Thus, the continued brain activation after creative task cessation may reflect these post-task emotional states.

Based on the observed associations among brain activation, autonomic nervous system indices, and VAS scores during and after the task, the findings indicate that the creative task elicited positive emotions, which in turn influenced frontal pole activation and autonomic nervous activity.

### Limitations and future directions

First, the number of participants was small. Calculated using G*Power software (Faul et al., 2009), thirty-four participants are required when comparing two conditions with α = 0.05, p-value < 0.05, effect size of 0.5, and a power of 0.8. However, here, the number of participants was 23. Thus, future studies with larger sample size are required to consolidate the results of this study. However, during and post-task statistical powers, were significantly different for the Oxy-Hb increase between both task conditions. The statistical power was approximately 0.8 during the task and over 0.8 in the post-task period; therefore, we considered that the main results of the study are not affected by the small sample size. Second, in this study, as an HRV index, only the LF/HF rate was analyzed, and caution is needed when interpreting the readings of HRV indices. Third, clearly separating emotion-induced brain activity from cognition-induced activity is challenging because of the common neural basis between the emotion induced by self-relatedness and the cognitive factor of creative thinking.

As a future direction, to assess the stress state during the creative activity, adopting some HRV indices other than the LF/HF ratio among multiple HRV indices is better (Shaffer and Ginsberg, 2017). Furthermore, tasks that can discriminate the activity of the frontal pole derived from cognitive functions (stimulus-oriented cognition) and emotions elicited by self-relevance (stimulus-independent introspection) need to be created.

## Conclusion

Using NIR-TRS, we measured the frontal pole activation induced by a positive emotion during a creative task using a real object. Herein, during and post-task brain activation was greater when performing the given creative task compared with the simple task. Brain activation in the latter half (through the end) of the creative task was related to the heart rate and stress indices, suggesting that the brain activation in this period was not due to sympathetic hyperactivity. Furthermore, when performing the creative task, the Oxy-Hb increase occurring immediately after end of the task (for 1 min duration) showed significant correlations with subjective investigation scores reflecting enjoyment. Thus, it reflects positive emotions. NIR-TRS is a good method for detecting changes in cerebral activation caused by emotions during tasks using real objects under natural settings. This study paves way for objective evaluation of changes in psychological states during task performance in people who have difficulty expressing their emotional states due to symptoms, such as cognitive decline or apathy, typically, patients with dementia or psychiatric disorders like depression, or alexithymia.

## CRediT authorship contribution statement

**Yumi Oboshi:** Writing - review & editing, Writing original draft, Investigation, Formal analysis, Conceptualization. **Kazuki Tamura:** Writing - original draft, Investigation, Formal analysis. **Yasuko Fukushi:** Writing - original draft, Investigation, Conceptualization. **Seiji Yamamoto:** Writing - review & editing, Project administration, Funding acquisition, Conceptualization.

## Declaration of Competing Interest

The authors declare that they have no known competing financial interests or personal relationships that could have appeared to influence the work reported in this paper.

The authors have read and have abided by the statement of ethical standards for manuscripts submitted to IBRO Neuroscience Reports.

The authors declare that all data acquisition on human subjects were conducted in accordance with the Declaration of Helsinki and that all procedures were carried out with the adequate understanding and written consent of the subjects.

We certify that formal approval to conduct the experiments described has been obtained from the human subjects review board, The Ethics Committee of Hamamatsu University School of Medicine (Hamamatsu, Japan), and could be provided upon request.

## Data Availability

The dataset analyzed in this study is available from the corresponding author upon reasonable request.
